# Bioassay-Guided Isolation of Cytotoxic Isocryptoporic Acids from *Cryptoporus volvatus*

**DOI:** 10.3390/molecules21121692

**Published:** 2016-12-08

**Authors:** Ling-Yun Zhou, Xiao-Hong Yu, Bin Lu, Yan Hua

**Affiliations:** 1 Key Laboratory for Forest Resources Conservation and Use in the Southwest Mountains of China, Southwest Forestry University, Kunming 650224, China; zly4321@sina.com (L.-Y.Z.); 13187815521@163.com (X.-H.Y.); nongxue022@163.com (B.L.); 2 School of Pharmacy, Wannan Medical College, Wuhu 241002, China

**Keywords:** *Cryptoporus volvatus*, isocryptoporic acids, cytotoxic activities against cancer cell lines

## Abstract

The present work constitutes a contribution to the phytochemical investigation of *Cryptoporus volvatus* aiming to search for effective cytotoxic constituents against tumor cell lines in vivo. Bioassay-guided separation of the ethylacetate extract of *C. volvatus* afforded four new isocryptoporic acid (ICA) derivatives, ICA-B trimethyl ester (**1**), ICA-E (**2**), ICA-E pentamethyl ester (**3**), and ICA-G (**4**), together with nine known cryptoporic acids. These isocryptoporic acids are isomers of the cryptoporic acids with drimenol instead of albicanol as the terpenoid fragment; their structures were elucidated on the basis of spectroscopic evidences (UV, IR, HRMS, and NMR) and comparison with literature values. All isolates show certain cytotoxic activities against five tumor cell lines. Among them, compound **4** showed an comparable activity to that of the positive control *cis*-platin, while other compounds exhibited weak cytotoxic activities.

## 1. Introduction

*Cryptoporus volvatus* (Pk.) Hubbard, belonging to the Polyporaceae, grows on living trees or dead wood. The fruiting bodies of this fungus have long been used for the treatment of asthma and bronchitis in Chinese traditional medicine [1]. In previous reports, several cryptoporic acid (CA) derivatives have been isolated from this plant [2,3,4,5]. Pharmacological investigations have indicated that this type of compound has strong superoxide-release inhibition [3] and antitumor-promotion properties [6]. In order to find other constituents with higher cytotoxic activities, we examined the fruiting bodies of *C. volvatus* by the bioassay-guided separation, which led to the isolation of four new chemical components as well as nine known components (CA-A, CA-A trimethyl ester, CA-B, CA-B trimethyl ester, CA-C, CA-C pentamethyl ester, CA-D, CA-D pentamethyl ester, and CA-E pentamethyl ester). In this paper, the isolation, structural elucidation, and cytotoxic properties towards five human cancer cell lines of all fractions and isolates were reported.

## 2. Results and Discussion

### 2.1. Identification of New Compounds

**Figure 1:**
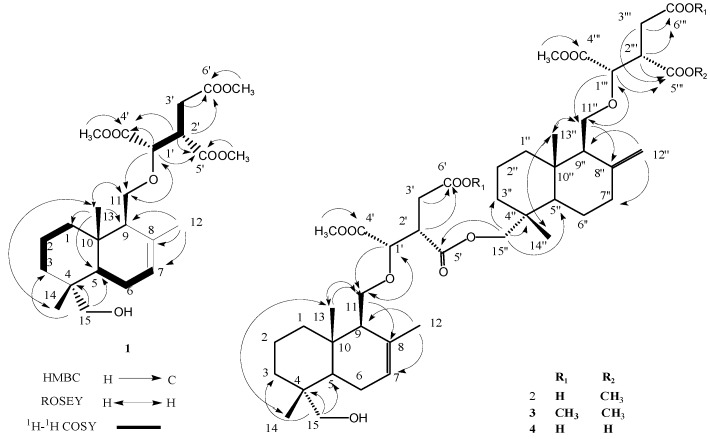
The structure of compounds **1**–**4**.

Compound **1** was obtained as a yellow oil and the molecular formula was established by MALDI-TOF/TOF-MS as C_24_H_38_O_8_, with a quasi-molecular ion peak of [M + Na]^+^ at *m*/*z* 477.2464 (calcd. for C_24_H_38_O_8_Na, 477.2459). The ^1^H-NMR spectrum (Table 1) showed two quaternary methyls at δ_H_ 0.83 and 0.84, a broad singlet at 5.43 ppm characteristic of a double bond, a methyl group at δ_H_ 1.74 attached to a double bond, and six signals between 2.5 and 4.1 ppm, indicative of the presence of heteroatoms in the molecular structure. The ^13^C-NMR spectrum (Table 2) revealed the presence of a double bond with carbon chemical shifts at δ_C_ 122.5 and 133.4, two carbons attached to oxygen, at δ_C_ 72.0 (CH_2_) and 78.7 (CH), and three carbonyl carbons at δ_C_ 170.8, 171.1, and 172.2. The NMR data for **1** were similar to that of isocryptoporic acid H (ICA-H) [7], expect for the presence of a hydroxymethyl group in place of one tertiary methyl group in ICA-H, indicating that one of the three tertiary methyl groups of ICA-H was replaced by a hydroxymethyl group. The additional hydroxyl group was found to be linked to C-15, which was determined by the presence of the NOE between H-5 and H-15 as well as HMBC correlations of δ_H_ 1.33 and 1.45 (2H, m, H-3), 0.84 (3H, s, H-14), and δ_C_ 72.0 (C-15). The planar structure of **1** was confirmed by analyses of ^1^H-^1^H COSY, HSQC, and HMBC data, where a drimenol instead of an albicanol moiety of cryptoporic acid B trimethyl ester [3] is present in the molecule structure. The relative configuration of **1** was established by NOESY analyses, where the cross-peak network observed between 11-CH_3_/13-CH_3_/14-CH_3_/1′-CH confirmed that they were located on the same ring face. The absolute configurations of the isocitric acid moiety were proposed to be either (1′R, 2′S) or (1′S, 2′R) by comparing ^1^H-NMR spectral analysis (4.09 (H-1′) and 3.45 (H-2′)) to the four synthesized diastereoisomericcryptoporic acid [8]. Comparison of the optical rotation with that of cryptoporic acid B trimethyl ester [3], the absolute configuration of **1** were finally confirmed as 1′R, 2′S (Figure 1). Compound **1**was then named isocryptoporic acid trimethyl ester (ICA-B trimethyl ester).

Compound **2** was obtained as a yellow oil. The molecular formula was established as C_45_H_68_O_15_ on the basis of MALDI-TOF/TOF-MS, which showed a quasimolecular-ion peak ([M + Na]^+^) at *m*/*z* 871.4446. The ^1^H-NMR and ^13^C-NMR spectra (Table 1 and Table 2) showed that the molecular contained four quaternary methyls, one methyl group attached to a double bond, three methoxyls, one exocyclic methylene, three methylenes bearing an oxygen, four ester carbonyls, and two carboxylic groups (Table 1 and Table 2). The ^1^H-^1^H and ^13^C-^1^H 2D COSY spectra of **2** displayed the presence of signals corresponding to ICA-B and CA-B, indicating that compound **2**, similar to CA-E [3], is the dimer of two subunits, esterified between the hydroxyl group at C-15 of CA-B and one of the carboxylic acid groups in the isocitric moiety of ICA-B. The position of two carboxylic acids at C-3′ and C-3′′′ and of dimerization at C-5′ with the primary alcohol of ICA-B were further established by the 2D NMR spectra of **2**. The relative and absolute configurations of C-1′, C-1′′′, C-2′, and C-2′′′ in the isocitrate moieties were determined to be (1′R, 1′′′R, 2′S, 2′′′S), in the same way described for compound **1**. On the basis of the above data, the structure of **2** was determined and named isocryptoporic acid E (ICA-E).

Compound **3** was isolated as a yellow oil. Its molecular formulaC_47_H_72_O_15_ was inferred from the MALDI-TOF/TOF-MS ([M + Na]^+^) at *m*/*z* 899.4776, 28 mass more than that of **2**, which suggests that **3** should be anester derivant of **2**. In addition, the ^1^H- and ^13^C-NMR spectra of **3** further confirmed the deduction, which were quite similar to those of **2**, except for the additional signal of two methoxyl groups. Furthermore, two carboxylic groups at C-3′ and C-3′′′ esterified into two methyl esters were indicated by ^13^C-^1^H 2D COSY and HMBC spectra. The absolute configuration of **3** was established as that of **2**, which was identical to that of **2**. Accordingly, the structure of **2** was assigned as shown and was named ICA-E pentamethyl ester.

Compound **4** was assigned the molecular formula C_44_H_66_O_15_ by analysis of its ^13^C-NMR and MALDI-TOF/TOF-MS (*m*/*z* 833.4343, [M − H]^+^). Expect for the absence of one methoxyl group, the ^1^H- and ^13^C-NMR spectra (Table 1 and Table 2) were very close to those of **2**, indicating that **3** is a monode-methyl derivative of **2**. The position of being demethyled was confirmed for the methyl ester at C-2′′ by the HMBC cross-peaks MeO-C(4′)/C-4′, MeO-C(4′′)/C-4′′, H-C(2′)/C-4′, C-5′, and C-6′. In fact, the structure of **4** is the isomer of the known compound CA-G. According to the previous judgment method, the absolute configuration of **4** presented the same conclusion as that of the above compounds. Thus, the structure was finally characterized to be **4** and named isocryptoporic acid G (ICA-G).

### 2.2. Cytotoxic Activities

The cytotoxic activities of different crude extracts (see Table 3) were assessed in vitro by the MTS cell proliferation assay against a panel of five human cancer cell lines. The activity of the EtOAc extract was similar to that of the MeOH extract, but the extraction rate was higher. In addition, both these extracts displayed better cytotoxic activity than the 60% EtOH extract. Therefore, we finally used ethyl acetate as the extraction solvent. The EtOAc extract was chromatographed on silica gel CC to yieldeight fractions. All fractions were further screened via MTS assay. Fraction 1 and Fraction 2, containing a large number of fatty acids and cholesterols, showed weak cytotoxic activities, while Fractions 3–6 with certain cytotoxic activities were finally proved to be consisted mostly of sesquiterpene acids, one of which had been reported to have certain cytotoxic activities. With the eluent polarity increased, more pigment-like impurities were eluted. As a result, the cytotoxic activities of these elutionswere decreased. Further bioassay-guided separation of Fractions 3–6 yielded 13 sesquiterpene acids. All isolates were tested for their cytotoxic activity against five human cancer cell lines. *Cis*-platin was a broad spectrum first-line anti-tumor drug and was used as a positive control. Among these compounds, compound **4** showed comparable activity to that of the positive control *cis*-platin (see Table 4), while other compounds exhibited weak cytotoxic activities with IC_50_ values of 50~100 μM (data not shown). It is concluded that the ratio of carboxyl groups and ester groups may play an important role in attaining better and more selective activity. Further analysis of the structure–activity relationship requiresmore compounds of this type.

## 3. Experimental Section

### 3.1. General Procedures

The optical rotations of the compounds were measured with a JASCO P-1020 DIP digital polarimeter (Jasco, Tokyo, Japan). The IR spectra were recorded on a Bruker Tensor-27 spectrometer with KBr pellets (Bruker, Bremen, Germany). The UV spectra were recorded on a Shimadzu UV-2401 PC spectrometer (Shimadzu, Suzhou, China). The 1D- and 2D-NMR spectra were obtained using an Avance III-500 instruments (Bruker) with TMS as the internal standard. The ESI-MS experiment was performed on Bruker HTC/Esquire mass spectrometer. MADLI-TOF/TOF-MS was carried out with an Bruker Daltonics Flex Analysis spectrometer (Bruker). An Agilent 1100 series machine (NO. 2008052910000035, Agilent, Beijing, China) equipped with a semi-preparative Agilent Zorbax SB-C18 column (9.4 mm × 250 mm, 5 mm, Beijing, China) was used for sample preparation. Column chromatography was performed using silica gel (200–300 mesh, Qingdao Haiyang Chemical Co., Ltd., Qingdao, China), Sephadex LH-20 (GE Healthcare Bio-Sciences AB, Fairfield, CT, USA), and reversed-phase C18 silica gel (50 mm, Merck, Darmstadt, Germany). Analytical and preparative TLC were performed using precoated GF_254_ plates (0.25 mm thickness, Qingdao Haiyang Chemical Co., Ltd., Qingdao, China). The detection was performed by spraying the plates with 5% sulfuric acid followed by heating.

### 3.2. Plant Material

The fruiting bodies of *Cryptoporus volvatus* (Basidiospores dimensions: 10~11 μm × 5~5.5 μm) was collected in Lijiang, Yunnan Province, China, in April 2015 and identified by Doctor Ying Zhang of Southwest Forestry University, a specialist in the field of fungal identification. A voucher specimen (No. YK01) is deposited at College of Forestry, Southwest Forestry University, Kunming, China.

### 3.3. Extraction and Isolation

The fruiting bodies of *C. volvatus* (10 kg) weredried by the sun and then cut with aknife. Small pieces of the material were extracted three times with EtOAc (15 L each time) under 60 °C for 2 h, and the combined solution was concentrated to produce a crude extract (2.1 kg). The extract was chromatographed on silica gel (5 kg) using a petroleum ether–ethylacetate gradient (1:0 to 0:1) to yield 8 fractions (Fractions 1~8). Fraction 1 (300 g) was composed of fatty acids. Fraction 2 (250 g) consisted of ergosterol derivatives. Fraction 3 (200 g) was further chromatographed on silica gel (1 kg) using petroleumether–ethylacetate (8:1 to 4:1) to furnish compound **1** (15 mg). Fraction 4 (100 g) was subjected to further silica gel CC, eluted with petroleumether–acetone (6:1 to 1:1) to afford four fractions (Fractions 4-A~D). Fraction 4-C (10 g) was rechromatographed on Sephadex LH-20 CC (CHCl_3_–EtOH = 7:3) to affordthe mixtures (4 g) of drimane-type sesquiterpenoids, which were further purified by semi-HPLC (CH_3_CN:H_2_O = 65:35, λ = 210 nm) to produce compound **2** (12.5 mg, Retention time = 8.5 min) and compound **3** (10 mg, Retention time = 10.3 min). Fraction 5 (150 g) was fractioned by MCI gel CC, eluted with MeOH–H_2_O (6:4 to 1:0) to yield 3 fractions (Fractions 5-A~D). Fraction 5-B (10 g) was purified using silica gel CC and eluted with petroleumether–acetone (2:1 to 1:2) to yield compound **4** (14 mg).

*Isocryptoporic acid B trimethyl ester* (**1**). Yellow oil; UV (MeOH) ν_max_ (log ε): 204 (0.27) nm; ${\left[\alpha \right]}_{\text{D}}^{20}$ +36 (*c* 2.10, MeOH); IR: 3431, 2950, 2929, 1742, 1663, 1044 cm^−^^1^; ^1^H-NMR (500 MHz, CHCl_3_) and ^13^C-NMR (125 MHz, CHCl_3_) data, see Table 1; MALDI-TOF/TOF-MS (*m/z*): 477.2464 [M + Na]^+^ (calcd. for C_24_H_38_O_8_Na, 477.2459).

*Isocryptoporic acid E* (**2**). Yellow oil; UV (MeOH) ν_max_ (log ε): 203 (0.26) nm; ${\left[\alpha \right]}_{\text{D}}^{20}$ +23 (*c* 2.20, MeOH); IR: 3431, 2946, 2929, 1739, 1640, 1040 cm^−^^1^; ^1^H-NMR (500 MHz, CHCl_3_) and ^13^C-NMR (125 MHz, CHCl_3_) data, see Table 1; MALDI-TOF/TOF-MS (*m/z*): 871.4446 [M + Na]^+^ (calcd. for C_45_H_68_O_15_Na, 871.4439).

*Isocryptoporic acid E pentamethyl ester* (**3**). Yellow oil; UV (MeOH) ν_max_ (log ε): 203 (0.22) nm; ${\left[\alpha \right]}_{\text{D}}^{20}$ +24 (*c* 2.20, MeOH); IR: 3433, 2945, 2930, 1740, 1644, 1044 cm^−^^1^; ^1^H-NMR (500 MHz, CHCl_3_) and ^13^C-NMR (125 MHz, CHCl_3_) data, see Table 1; MALDI-TOF/TOF-MS (*m/z*): 899.4776 [M + Na]^+^ (calcd. for C_47_H_72_O_15_Na, 899.4763).

*Isocryptoporic acid G* (**4**). Yellow oil; UV (MeOH) ν_max_ (log ε): 203 (0.35) nm; ${\left[\alpha \right]}_{\text{D}}^{20}$ +21 (*c* 1.70, MeOH); IR: 3426, 2946, 2929, 1739, 1638, 1034 cm^−^^1^; ^1^H-NMR (500 MHz, CHCl_3_) and ^13^C-NMR (125 MHz, CHCl_3_) data, see Table 1; MALDI-TOF/TOF-MS (*m/z*): 833.4343 [M − H]^+^ (calcd. for C_44_H_66_O_15_, 833.4329).

### 3.4. Assays of Cytotoxic Activity

Five human cancer cell lines, i.e., lung cancer (A-549), human myeloid leukemia (HL-60), breast cancer (MCF-7), hepatocellular carcinoma (SMMC-7721), and colon cancer (SW-480) were purchased from American Type Culture Collection (ATCC) (Manassas, VA, USA) and cultured in RPMI 1640 medium (HL-60 and SMMC-7721) or DMEM medium (A-549, MCF-7and SW-480). The cytotoxic activities of tumor cell lines were performed in vitro by the MTS cell proliferation assay. Briefly, the cells were seeded at a concentration of 5 × 10^3^ cell/mL in a volume of 0.1 mL in 96-well plates and incubated for 24 h at 37 °C in a CO_2_ incubator. Test sample portions of 100 μL at varied concentrations, prepared in culture medium, were added to each well. After 48 h of incubation at 37 °C in the CO_2_ incubator, the cells in each well were incubated in culture medium with 20 μL of a 5 M solution of MTS, which was purchased from Promega Biotechnology Corporation (Beijing, China) and dissolved in PBS (pH = 6.0), for 1 h at 37 °C. After another hour of incubation, absorption was measured at 490 nm via MULTISKAN FC. All experiments were carried out in triplicate. The cytotoxicity of each sample was expressed as IC_50_. The inhibition ratio of cells proliferation was calculated as follows [9]:

Inhibition ratio (%) = (1 − (OD − OD_blank_)/(OD_0_ − OD_blank_)) × 100% (1)

where OD, OD_0_, and OD_blank_ are the absorbance of treated cells, untreated cells, and the blank control, respectively.

## 4. Conclusions

In this study, the EtOAc extract of *Cryptoporus volvatus* (Pk.) Hubbard was chromatographed to get different fractions. On the basis of the evaluation in vitro cytotoxic activity using the MTS assay to all fractions, the bioassay-guided separation led to the isolation of four new isocryptoporic acid derivatives, together with nine known cryptoporic acids. This kind of isocryptoporic acid was reported in this genus for the first time.In addition, the activity data indicated that all compounds had certain inhibitory activities against five cancer cells. Compound **4** especially had moderate inhibitory activities, exhibiting close inhibitions with the positive control *cis*-platin.

## Figures and Tables

**Table 1:** The ^1^H-NMR spectral data for compounds **1**–**4** (500 MHz, CDCl_3_).

H	1	2	3	4
1	1.03 (m), 1.93 (m)	1.03 (m), 1.88 (m)	1.03 (m), 1.84 (m)	1.03 (m), 1.88 (m)
2	1.51 (m), 1.62 (m)	1.50 (m), 1.63 (m)	1.48 (m), 1.63 (m)	1.50 (m), 1.63 (m)
3	1.33 (m), 1.45 (m)	1.34 (m), 1.42 (m)	1.33 (m), 1.42 (m)	1.32 (m), 1.42 (m)
5	1.48 (m)	1.52 (m)	1.52 (m)	1.53 (m)
6	1.82 (m)	1.88 (m)	1.86 (m)	1.86 (m)
7	5.43 (brs)	5.42 (brs)	5.41 (brs)	5.44 (brs)
9	1.95 (m)	1.94 (m)	1.93 (m)	1.94 (m)
11	3.43 (dd, 9.8, 3.2)	3.46 (m)	3.43 (m)	3.48 (m)
	3.80 (dd, 9.8, 6.0)	3.84 (m)	3.86 (m)	3.82 (m)
12	1.87 (brs)	1.73 (brs)	1.70 (brs)	1.75 (brs)
13	0.83 (s)	0.84 (s)	0.83 (s)	0.84 (s)
14	0.84 (s)	0.84 (s)	0.84 (s)	0.85 (s)
15	3.11 (d, 10.8)	3.12 (d, 11.2)	3.11 (d, 11.2)	3.12 (d, 11.2)
	3.35 (d, 10.8)	3.35 (d, 11.2)	3.35 (d, 11.2)	3.37 (d, 11.2)
1′	4.09 (d, 4.2)	4.10 (d, 4.8)	4.08 (d, 4.8)	4.11 (d, 4.8)
2′	3.45 (m)	3.41 (m)	3.43 (m)	3.43 (m)
3′	2.53 (dd, 16.8, 4.4)	2.64 (m)	2.55 (m)	2.64 (m)
	2.80 (dd, 16.8, 9.2)	2.82 (m)	2.78 (m)	2.80 (m)
1′′		1.13 (m), 1.66 (m)	1.15 (m), 1.68 (m)	1.12 (m), 1.65 (m)
2′′		1.52 (m), 1.63 (m)	1.50 (m), 1.63 (m)	1.50 (m), 1.66 (m)
3′′		1.21 (m), 1.58 (m)	1.23 (m), 1.58 (m)	1.20 (m), 1.58 (m)
5′′		1.34 (m)	1.31 (m)	1.35 (m)
6′′		1.35 (m), 1.57 (m)	1.33 (m), 1.55 (m)	1.34 (m), 1.55 (m)
7′′		2.00 (m), 2.38 (m)	2.01 (m), 2.36 (m)	2.01 (m), 2.38 (m)
9′′		1.99 (m)	1.96 (m)	2.03 (m)
11′′		3.53 (m)	3.51 (m)	3.55 (m)
		3.92 (m)	3.89 (m)	3.94 (m)
12′′		4.78 (brs), 4.84 (brs)	4.75 (brs), 4.84 (brs)	4.78 (brs), 4.86 (brs)
13′′		0.74 (s)	0.75 (s)	0.75 (s)
14′′		0.75 (s)	0.74 (s)	0.76 (s)
15′′		3.62 (d, 11.2)	3.65 (d, 11.2)	3.45 (d, 11.2)
		4.06 (d, 11.2)	3.86 (d, 11.2)	3.96 (d, 11.2)
1′′′		4.14 (d, 4.8)	4.10 (d, 4.8)	4.18 (d, 4.8)
2′′′		3.40 (m)	3.42 (m)	3.38 (m)
3′′′		2.72 (m)	2.53 (m)	2.60 (m)
		2.75 (m)	2.78 (m)	2.68 (m)
OMe	3.65 (s)	3.68 (s)	3.67 (s)	3.75 (s)
	3.65 (s)	3.76 (s)	3.67 (s)	3.76 (s)
	3.68 (s)	3.76 (s)	3.68 (s)	
			3.74 (s)	
			3.75 (s)	

**Table 2:** The ^13^C-NMR spectral data for compounds **1**–**4** (125 MHz, CDCl_3_).

C	1	2	3	4
1	39.1	38.8	39.0	38.7
2	18.1	18.5	18.3	18.5
3	35.5	35.4	35.4	35.3
4	35.5	37.4	37.4	37.8
5	44.5	44.6	44.6	44.7
6	23.4	23.4	23.4	23.4
7	122.5	122.5	122.5	123.0
8	133.4	133.2	133.4	132.9
9	54.8	54.9	54.7	55.7
10	37.5	38.5	38.5	38.5
11	69.5	68.9	69.7	68.4
12	21.7	21.8	21.8	21.8
13	15.2	15.7	15.7	15.7
14	17.7	17.6	17.5	17.6
15	72.0	71.8	71.9	71.5
1′	78.7	78.4	78.5	77.7
2′	43.3	43.2	43.2	43.4
3′	31.9	32.0	32.1	32.3
4′	171.1	171.1	171.2	172.6
5′	170.8	170.7	170.8	170.8
6′	172.2	175.0	172.2	174.9
1′′		38.5	38.5	38.5
2′′		18.1	18.0	18.4
3′′		35.3	35.6	35.3
4′′		37.6	37.1	37.8
5′′		48.9	48.9	48.3
6′′		23.6	23.6	23.6
7′′		36.8	36.7	36.8
8′′		146.3	146.3	146.5
9′′		55.1	54.8	55.3
10′′		38.9	39.0	38.7
11′′		68.2	68.2	68.4
12′′		108.2	108.2	108.2
13′′		15.6	15.6	15.6
14′′		17.7	17.6	17.7
15′′		73.6	73.8	73.2
1′′′		78.8	78.9	78.8
2′′′		44.6	44.4	44.7
3′′′		31.7	31.7	32.0
4′′′		170.9	170.8	171.1
5′′′		171.1	170.7	175.0
6′′′		175.2	172.1	176.0
OMe	52.0	52.1	51.9	52.2
	52.0	52.2	51.9	52.2
	52.2	52.2	52.0	
			52.0	
			52.0	

**Table 3:** Cytotoxic activities of column chromatography fractions.

Samples	IC_50_ (μg/mL)				
	HL-60	A-549	SMMC-7721	MCF-7	SW480
The EtOAc extract	>200	>300	>300	>300	>300
The MeOH extract	>200	>300	>300	>300	>300
The 60% EtOH-H_2_O extract	>300	>500	>400	>400	>400
Fraction 1	>300	>400	>400	>400	>400
Fraction 2	>200	>300	>200	>200	>200
Fraction 3	80.81 ± 1.90	>100	95.19 ± 2.80	>100	87.82 ± 0.50
Fraction 4	70.22 ± 1.70	>100	88.97 ± 1.54	92.14 ± 1.90	80.13 ± 3.30
Fraction 5	67.35 ± 1.30	94.28 ± 1.35	85.53 ± 0.98	90.62 ± 1.50	85.67 ± 1.30
Fraction 6	80.87 ± 2.90	>100	>100	>100	97.14 ± 1.80
Fraction 7	98.26 ± 1.90	>100	>100	>100	>100
Fraction 8	>100	>300	>200	>200	>200

**Table 4:** Antitumor activities of compound **4** and the positive control *cis*-platin.

Compounds	IC_50_ (μM)				
	HL-60	A-549	SMMC-7721	MCF-7	SW480
**4**	20.92 ± 1.20	26.91 ± 1.80	21.19 ± 2.10	26.62 ± 1.60	17.82 ± 0.70
*Cis*-platin	1.68 ± 0.22	25.22 ± 1.15	14.85 ± 0.82	11.77 ± 2.28	4.28 ± 1.51
